# 

**Published:** 1907-10-26

**Authors:** 


St. Bartholomew's Hospital.—A Conversazione was given on the evening of the 23rd inst. by the medical staff and lecturers of the Medical School of St. Bartholomew's Hospital, in the new out-patient and special department block, recently opened by His Royal Highness the Prince of Wales. There was a large and distinguished gathering of guests, who were received by the Senior Physician and the Senior Surgeon to the Hospital, and afterwards distributed themselves over the fine and spacious halls and rooms of which the building consists. The ground floor is chiefly occupied by a lofty, well ventilated, and thoroughly serviceable waiting hall, beautifully decorated for the occasion with flowers. Here the band of the 1st Life Guards gave selections of music throughout the evening. In the medical waiting room was shown a unique collection of old prints and other objects of historical and personal interest associated with the long past of the Hospital. It is needless to say that this attracted great attention. In the surgical consulting room, the finest of its kind in London, an exhibition was given illustrating the important part that pathology takes in the service of the sick poor. On the next floor the nursing staff of the Hospital demonstrated how their wise minds and deft fingers could do much to ease the irksomeness of a sick bed. The fine dispensary and pharmaceutical laboratory, and the hospital kitchen were duly inspected. The visitors were shown how the actual meals of the patients were cooked and sent on their way to the wards. Many partook of soup from one of the huge cauldrons. So beautifully balanced was this cavernous receptacle that a cupful of steaming liquid could be poured through its enormous lip by a mechanism so simple that even a child could work it. It was long past midnight when the last of the carriages rolled away, and everyone who had been present agreed that a new and most propitious era had dawned upon the Royal Hospital of St. Bartholomew, which had thrown open its doors to suffering humanity upon that very site for upwards of eight hundred years.

